# Effects of vitamin D supplementation on liver fibrogenic factors, vitamin D receptor and liver fibrogenic microRNAs in metabolic dysfunction-associated steatotic liver disease (MASLD) patients: an exploratory randomized clinical trial

**DOI:** 10.1186/s12937-024-00911-x

**Published:** 2024-02-27

**Authors:** Soraiya Ebrahimpour-Koujan, Amir Ali Sohrabpour, Edward Giovannucci, Akram Vatannejad, Ahmad Esmaillzadeh

**Affiliations:** 1 Department of Community Nutrition, School of Nutritional Sciences and Dietetics, Tehran University of Medical Sciences, PO Box 14155-6117, Tehran, Iran; 2 Department of Clinical Nutrition, School of Nutritional Sciences and Dietetics, Tehran University of Medical Sciences, Tehran, Iran; 3 The Liver, Pancreatic, and Biliary Disease Research Center, Digestive Disease Research Institute, Shariati Hospital, Tehran University of Medical Sciences, Tehran, Iran; 4 Departments of Nutrition and Epidemiology, Harvard T.H. Chan School of Public Health, Boston, MA USA; 5 Department of Comparative Biosciences, Faculty of Veterinary Medicine, University of Tehran, Tehran, Iran; 6 Obesity and Eating Habits Research Center, Endocrinology and Metabolism Molecular -Cellular Sciences Institute, Tehran University of Medical Sciences, Tehran, Iran; 7 Department of Community Nutrition, School of Nutrition and Food Science, Isfahan University of Medical Sciences, Isfahan, Iran

**Keywords:** Metabolic dysfunction-Associated Steatotic Liver Disease, Fibrogenic factor, MicroRNA, Vitamin D, Clinical trial

## Abstract

**Background and aims:**

Metabolic dysfunction-associated steatotic liver disease (MASLD) is a global metabolic problem which can lead to irreversible liver fibrosis. It has been shown that vitamin D and its receptors contribute to fibrogenic pathways in the liver. However, the effect of vitamin D supplementation on liver fibrosis related factors have not been examined. This double blinded placebo controlled clinical trial was designed to investigate the effects on vitamin D supplementation on serum levels of VDR, fibrogenic factors and fibrogenic MicroRNAs in MASLD patients.

**Methods:**

Forty six MASLD patients after block matching for sex and BMI were randomly assigned to receive 4000 IU/d vitamin D or placebo for 12 weeks. Weight, height and waist circumference were measured. Serum fibrogenic microRNAs, laminin, collagen type IV, hyaluronic acid, vitamin D, VDR, PTH, blood fasting glucose, serum fasting insulin, lipid profile, ALT and AST were determined at the baseline and at the end of the trial. Insulin resistance and insulin sensitivity were calculated using the HOMA-IR and QUICKI equation.

**Results:**

Supplementation with vitamin D for 12 weeks led to the significant increases in serum 25(OH) vitamin D, VDR and HDL-C compared to placebo (*P* < 0.001, *P* = 0.008 and *P* < 0.001). There were significant decreases in ALT, AST, FBS and LDL-C levels in the vitamin D group as compared to the placebo (*P* < 0.05). Laminin and hyaluronic acid concentrations were significantly decreased in the vitamin D group as compared to the placebo group, by -10.6 and − 28.7 ng/mL, respectively. Supplementation with vitamin D for 12 weeks resulted in a significant lower MiR-21 and MiR-122 gene expressions compared to the placebo group (*P* = 0.01 and *P* < 0.001, respectively).

**Discussion:**

As the first randomized controlled trial on the effect of vitamin D supplementation on serum levels of VDR, fibrogenic factors and fibrogenic MicroRNAs in MASLD patients, we found a significant reduction in some liver fibrogenic factors, in liver transaminases and corresponding changes in some fibrosis-related MiRs and some metabolic factors. Further clinical trials with larger sample sizes and direct measures of liver fibrosis are needed to confirm these findings.

**Trial registration number:**

(available at: http://www.irct.ir, identifier: IRCT201405251485N13), Registration date: 14-03-2017.

**Supplementary Information:**

The online version contains supplementary material available at 10.1186/s12937-024-00911-x.

## Introduction

Metabolic dysfunction-associated steatotic liver disease (MASLD) is a global metabolic problem, affecting around a quarter of the world’s adult population [1]. This condition represents a spectrum of chronic liver disease, beginning with simple steatosis that may progress to steatohepatitis, fibrosis, cirrhosis and hepatocellular carcinoma [2]. In the Middle-East, more than 32% of adult population are affected [1, 3]. Liver fibrosis results from chronic damage to the liver [4]. Production of some fibrogenic factors by hepatic stellate cells (HSCs) including collagens, laminin and hyaluronic acid were identified as major steps in the liver fibrosis in MASLD patients [5]. Studies suggest that liver fibrosis development can be inhibited by decreasing levels of fibrogenic factors [4]. No optimal therapeutic regimens exist for liver fibrosis [6]; however, combination of lifestyle modification, medications and probably dietary supplements might have favorable effects on regression of liver fibrosis [7, 8]. Recently, gene expression of specific microRNAs has attracted more attention toward liver fibrosis in MASLD patients [9, 10]. Along with contributing to fibrosis signaling, they also involved in lipid and cholesterol metabolism in the liver [11].

A growing body of evidence has indicated that vitamin D deficiency is highly prevalent among patients with MASLD [12]. Vitamin D has also been shown to contribute to poor outcome and progression to liver fibrosis [2, 13, 14]. Experimental studies have indicated that vitamin D and its receptor (VDR) are involved in suppressing fibro-genic signaling [15]. Earlier studies have shown that microRNAs and fibrogenic factors are main mediators in fibrosis progression [16, 17]. Based on experimental studies, some microRNAs including MiR-122, MiR-34a and MiR-21 are involved in liver lipid metabolism and normal turnover of hepatocytes [18]. Dysregulation of these microRNAs is considered to mediate of liver fibrosis [11, 19]. Previous clinical trials have mostly examined the effects of vitamin D supplementation on serum concentrations of inflammatory markers and lipid profiles in MASLD patients [20–23]. We are aware of no study of vitamin D supplementation investigating the effects on microRNAs and fibrogenic factors. This randomized, placebo-controlled, parallel clinical trial was therefore designed to examine the effects of vitamin D supplementation on serum levels of VDR, fibro-genic factors and fibrosis-related microRNAs in MASLD patients.

## Methods

### Participants

This parallel randomized double-blind placebo-controlled clinical trial (RCT) included patients with non-alcoholic steatohepatosis (NASH as confirmed by B-ultrasound and fibro-scan reports). We considered grades 2 and 3 of B-ultrasound reports and CAP score > 310 and fibrosis score > 6 of fibroscan reports as our objective criteria. Based on the suggested formula for parallel clinical trials and a possible drop out of 30%, given the type I error of 5% and study power of 90%, we reached the sample size of 23 patients in each group. The study was registered in the Iranian Registry of Clinical Trials website (available at: http://www.irct.ir, identifier: IRCT201405251485N13). Required and detailed information about study design and procedures used in the current study were previously published [24]. Briefly, we recruited MASLD patients with NASH, aged 20–60 year. Individuals with the following criteria were not included: smokers, those consuming alcohol, pregnant or lactating women or those who planned to get pregnant during the next 12 weeks, and those taking vitamin D supplements and antioxidants during the last 12 weeks. We also did not include individuals with some pathologic conditions affecting the liver, including viral hepatitis, any acute or chronic liver failure, and liver transplantation. All participants provided informed written consent.

### Study design

**Fig. 1:**
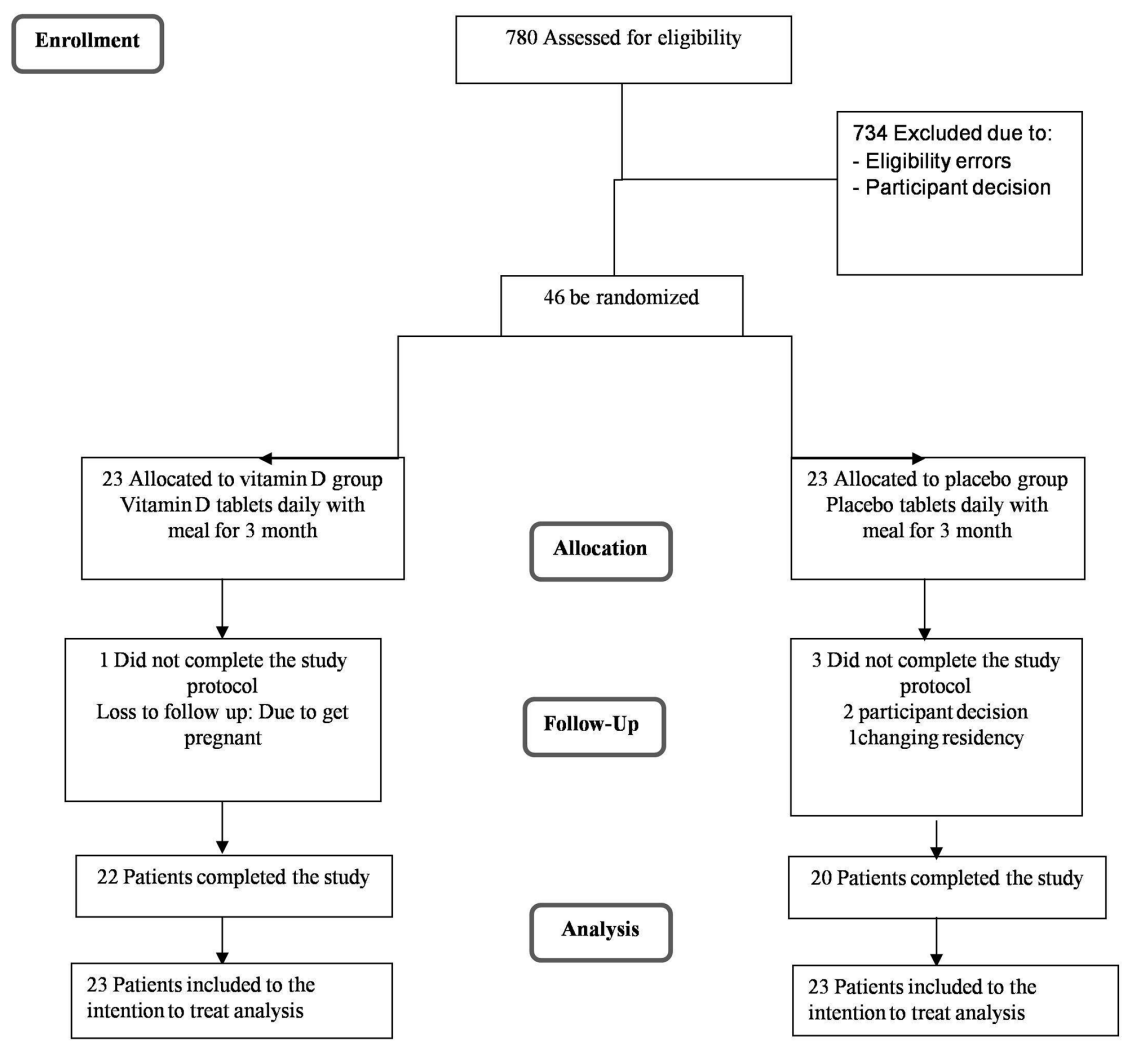
Flow diagram of the study participants

We conducted this trial in accordance with the guidelines laid down in the Declaration of Helsinki (1964). Forty six patients were enrolled in the whole trial. Prior to allocating participants into the vitamin D and placebo groups, anthropometric indices, including weight, height, waist circumference were measured, and BMI was calculated. Information about demographic characteristics, medical history and medication use as well as socio-economic status (SES) were collected at study baseline. Biochemical and molecular measurements were also done at study baseline and after 12-wk intervention. After these measurements, participants were randomly assigned to intervention and placebo groups based on block randomization method. Each block was composed of two persons with the same sex (male/female) and similar BMI (overweight/obese). Random allocation sequence was generated using Random Allocation Software: RAS [25]. A study diagram indicating individuals recruited in each group along with study design and dropouts is provided in Fig. 1.

### Intervention

All patients, researchers, the gastroenterologist, statistical analyst and laboratory staffs were blinded to the intervention. Based on earlier guidelines for treatment of vitamin D deficiency and insufficiency [26], we administered vitamin D at the dosage of 4000 IU per day for 12 weeks. Participants in the intervention group were requested to take vitamin D tablets daily with their main meals. Individuals in the placebo group were given an identical placebo tablets that contained lactose. Vitamin D and placebo tablets were produced by the PARS MINOO Co. Tehran, Iran. Vitamin D tablets and placebos were similar in terms of appearance, shape and odor. A third person that was not involved directly in the study packaged the vitamin D and placebo tablets in the bottles. The bottles were coded as A and B. The codes remained unknown to researchers until finishing the analyses. Patients took their bottles in two time periods: at their first visit and at the middle of the trial on week 6. To determine the adherence to the intervention, subjects were asked to record their daily consumption of supplements or placebos in a checklist given them by the investigators. To increase compliance and avoid forgetting the use of supplements and placebos, subjects received messages on their cell phones every day from the investigators’ side. In addition, we examined serum 25 (OH) vitamin D levels at study baseline and end of trial as a measure of compliance. To examine the possible toxicity and hypervitaminosis that might arise from taking vitamin D supplements, serum concentrations of PTH at study baseline and end of the trial were measured.

### Outcomes

The primary outcomes of the present clinical trial were levels of serum VDR, laminin, collagen type IV, hyaluronic acid, MiR-122, MiR-21 and MiR-34a. The secondary outcomes were liver enzymes, lipid profile and glycemic indices.

### Blood sampling, biochemical and molecular measurements

In a fasting state, a 10 mL venous blood sample was taken from each patient between 7:00 and 9:00 a.m. The samples were immediately centrifuged at 20 ^0^C, 3000 rpm for 10 min in aseptic condition. Serum samples separated in RNAase free micro-tubes in the clean room, where all equipments were UV exposed for 20 min. Serum lipid profiles (TC, HDL-C, LDL-C, TG), liver enzymes (ALT, AST) and glucose levels was measured by the enzymatic colorimetric method using PARS AZMOON kits. Then the samples stored at -70 ^0^C until measuring other biochemical and molecular factors. Serum insulin, vitamin D, VDR, PTH, laminin, collagen type IV and hyaluronic acid were all measured using enzyme-linked immunosorbent assay (ELISA) method. VDR, laminin, collagen type IV and hyaluronic acid was measured by Crystal Day kits. IBT, BIOMERICA and EUROIMMUN kits were used to measure serum levels of insulin, PTH and vitamin D, respectively. Insulin resistance and insulin sensitivity was determined using the HOMA-IR and QUICKI equations, respectively [27].

The gene expression of serum MicroRNAs (MiR-122, MiR-21 and MiR-34a) was determined by Real-time PCR method.

### RNA isolation and cDNA synthesis

Total RNA samples were isolated from serum samples using the mirVana miRNA isolation kit according to the manufacturer’s instructions (Ambion). The concentration and quantity of total RNA were measured at 260 and 280 nm (A260/280) using a NanoDrop 2000 spectrophotometer (NanoDrop Technologies). Total RNA (10 ng) from nonalcoholic fatty liver subjects were reverse transcribed into our specific target cDNA (u6 snRNA, MiR-122, MiR-21, MiR-34a) using specific primers and reagents provided by TaqMan® MicroRNA Assays (Applied Biosystems, Foster City, CA, USA) and Taqman® MicroRNA Reverse Transcription Kit (Applied Biosystems, Foster City, CA, USA) respectively.

### Quantitative real-time PCR

Real-time quantitative PCR was performed to measure the expression levels of microRNAs with the TaqMan® MicroRNA Assays (Applied Biosystems, Foster City, CA, USA), using probes for MiR-122-5p (assay ID: 002245), MiR-21-5p (assay ID: 000397), MiR-34a-5p (assay ID: 000426) and u6 snRNA housekeeping gene (assay ID: 001973), on a Step One Plus Real-Time PCR System (Applied Biosystems, Foster City, CA, USA). The data were normalized using u6 snRNA as endogenous control.

### Assessment of other variables

To examine dietary intakes and physical activity throughout the study, participants were asked to record their dietary intakes and physical activity in a day every 2 weeks. We had determined this specific day and recalled them in a regular order. Therefore, all patients provided 6 dietary records and 6 physical activity records (two for weekends and four for weekdays) during the study. We computed nutrient intakes of study participants based on the average of 6 dietary records using Nutritionist 4 software. To analyze physical activity records, we used MET-hours/d values for each physical activity, based on published guidelines [28], considering the time each participant spent on. Data on anthropometric measures, including body weight, height and waist circumference (WC) were collected according to standard protocols at study baseline and end of trial. BMI was computed as weight in kilogram divided by height in meters squared.

### Statistical analysis

Threshold cycle number was used to calculate the relative expression between samples. We used the ΔΔCt (cycle threshold) method in which relative expression = 2 ^−ΔΔCt^, where ΔΔCt = ∆CT intervention or placebo - ∆CT before mean of placebo [29]. The intention to treat approach was used for data analysis. In case of missing data at the endpoint, we used the mean of variables and replaced it for the endpoint values. Data presented as mean ± standard deviations (± SD). The one-sample Kolmogorov-Smirnov test was used for assessing the normality of the distribution of data. Repeated measure analysis of variance was used to identify the effect of the intervention of outcome variables. In addition, per-protocol analyses were also performed. ANCOVA analysis was used to adjust the confounding effects of baseline values and medications used in adjusted models. The changes of variables were determined by deducting the endpoint values from base line. The percentage of changes was computed by dividing the changes on base line values multiplied by 100. The correlations between changes of serum concentration vitamin D and changes of serum levels of fibrogenic factors and MIRs were assessed by Pearson coefficient test. *P* < 0.05 was considered as statistically significant. All analyses were done by blinded researcher prior to uncoding the study interventions. All statistical analyses of all data were performed using SPSS software version 21.0 (SPSS Inc., Chicago, IL, USA).

## Results

The trial was carried out between October 2018 and April 2019. The timing of recruitment, sampling, random allocation and treatment were all initiated in October 2018-January 2019 (during the cloudy/rainy season), and the final measurements after treatment were made in March-April 2019 (at the end of the cloudy/rainy season). Forty two patients (20 subjects in placebo group and 22 subjects in vitamin D group) completed the 12 weeks clinical trial. One patient in the vitamin D group became pregnant and was thereby excluded. Two patients in the placebo group moved to other city and one patient was a non-responder in the endpoint follow up. However, we performed intention-to-treat approach, and therefore data from all 46 patients were used in the analysis(Fig. 1). Participants did not report any adverse effects or symptoms with vitamin D supplementation during the trial and had good compliance with the intervention. Adherence rate was defined as taking ≥ 80% of assigned medication (vitamin D or placebo tablets). There was no significant difference between groups in terms of demographic characteristics, vitamin D deficiency, medication (except for statins) and dietary supplement use (*P* > 0.05) (Table 1). Means of anthropometric measures were not different as well comparing the two groups.

**Table 1:** General characteristics of MASLD patients at baseline

Variable	Placebo group (*n* = 23)	Vitamin D group (*n* = 23)	***P*** value^*^	
Age (y)	43.9 ± 11.0	47.3 ± 9.4	0.26	
Weight (kg)	89.3 ± 13.0	86.0 ± 17.8	0.47	
BMI (kg/m^2^)	33.0 ± 10.5	30.3 ± 4.4	0.26	
WC (cm)	109.7 ± 10.9	108.1 ± 12.3	0.65	
Female (%)	47.8	52.2	0.77	
Married (%)	82.6	87.0	0.53	
Educated (%)	95.7	95.7	0.99	
Vitamin D deficiency (< 30ng/ml) (%)	43.5	52.8	0.55	
MASLD grade (by B-ultrasound) (%)			0.54	
Grade 2	60.9	69.6		
Grade 3	39.1	30.4		
Medication use (%)				
NSAIDs	4.3	4.3	0.99	
Anti-acids	8.7	8.7	0.99	
Corticosteroids	4.3	0	0.31	
OHAs	13.0	21.7	0.44	
Livergol	8.7	26.1	0.12	
Statins^†^	13.0	39.1	0.04	
Ursodeoxycholic acid	0	8.7	0.15	
Anti-hypertensive	17.3	13.0	0.68	
Dietary supplement use (%)				
Vitamin E	8.7	17.4	0.38	
Iron-folate	8.7	0	0.15	
Calcium	4.3	0	0.31	
Multi vitamin	0	4.3	0.31	
Omega-3	4.3	0	0.31	

Values are presented as mean ± standard deviation except those are presented as percent

^*^Based on independent samples t-tests for quantitative variables and chi-square for qualitative variables

MASLD: Metabolic Dysfunction–Associated Liver Disease; BMI: Body Mass Index; WC: Waist Circumference

^†^Statistically significant differences between two groups was seen

Table 2 illustrates daily physical activity as well as dietary energy, food and nutrient intakes of MASLD patients throughout the 12 weeks of intervention. The comparison between groups showed that there were no significant difference in daily physical activity and dietary intakes.

**Table 2:** Physical activity and dietary intakes of participants throughout the study^†^

	Study baseline			Throughout the study		
	Placebo (*n* = 23)	Vitamin D (*n* = 23)	P*	Placebo (*n* = 23)	Vitamin D (*n* = 23)	P*
Physical activity (MET-h /day)	25.49 ± 18.9	23.13 ± 14.2	0.64	25.64 ± 11.2	20.35 ± 12.2	0.37
Total energy (kcal/d)	2376.8 ± 501.81	2583.7 ± 672.4	0.61	2545.7 ± 685.8	2855.6 ± 642.6	0.14
**Nutrients**						
Carbohydrates (g/day)	267.6 ± 128.7	303.5 ± 105.2	0.22	296.6 ± 108.7	330.3 ± 95.2	0.30
Total Protein (g/day)	95.1 ± 28.5	105.8 ± 29.13	0.74	96.5 ± 38.7	103.8 ± 28.1	0.49
Fats (g/day)	125.6 ± 48.1	97.8 ± 27.4	0.15	113.1 ± 37.7	130.4 ± 39.4	0.50
Total fiber (g/day)	15.14 ± 8.4	19.61 ± 11.8	0.11	15.9 ± 6.7	18.7 ± 10.3	0.16
Vitamin D (mg/day)	1.02 ± 1.24	1.09 ± 1.13	0.15	0.94 ± 1.17	0.99 ± 1.10	0.35
Vitamin E (mg/day)	4.22 ± 1.38	4.27 ± 1.78	0.88	3.42 ± 1.9	5.7 ± 6.1	0.89
Vitamin C (mg/day)	164.8 ± 91.5	178.8 ± 102.4	0.56	151.0 ± 85.5	169.2 ± 110.7	0.56
**Food groups**						
Fruit (g/day)	215.7 ± 19.4	236.1 ± 25.7	0.42	283.7 ± 176.4	268.2 ± 255.1	0.81
Vegetables (g/day)	314.7 ± 103.0	345.6 ± 111.7	0.78	356.5 ± 187.4	406.1 ± 304.8	0.54
Dairy (g/day)	135.9 ± 98.4	137.3 ± 101.0	0.95	198.6 ± 163.9	206.7 ± 171.7	0.88
Grains (g/day)	439.1 ± 215.7	482.2 ± 249.9	0.35	337.6 ± 160.5	359.8 ± 125.9	0.62
Red and processed meat (g/day)	67.3 ± 45.2	75.9 ± 55.9	0.43	74.7 ± 51.9	94.9 ± 65.0	0.28
Pasta (g/day)	19.6 ± 9.8	18.7 ± 10.2	0.66	14.1 ± 8.4	13.2 ± 5.9	0.71
Fish (g/day)	20.3 ± 14.1	20.1 ± 12.6	0.97	23.7 ± 19.1	21.1 ± 12.9	0.89
Egg (g/day)	26.7 ± 19.7	40.8 ± 27.5	0.06	53.9 ± 37.7	57.4 ± 39.1	0.35

*Obtained by Independent Sample t-test

MET: Metabolic Equivalents

^†^obtained by 6 physical activity and 24-hour food records

Metabolic profiles of patients at study baseline and after 12 weeks of intervention are shown in Table 3. At study baseline, there was no significant difference between the vitamin D and placebo groups in terms of all metabolic variables (*P* > 0.05). Supplementation with vitamin D for 12 weeks resulted in a clinically significant increase in serum 25(OH) vitamin D (changes from baseline in vitamin D group: 15.2 ng/mL vs. -3.9 ng/mL in placebo group, *P* < 0.001), VDR (4.1 ng/mL vs. -2.8 ng/mL, *P* = 0.008) and serum HDL-C concentrations (3.9 mg/dL vs. -2.0 mg/dL, *P* < 0.001) compared to placebo.

**Table 3:** Metabolic status of study participants at baseline and after 12 weeks trial

	Study Baseline			End of trial			Changes from baseline		
	Placebo Mean ± SD (CI)	Vitamin D Mean ± SD (CI)	***P*** ^*^	Placebo Mean ± SD (CI)	Vitamin D Mean ± SD (CI)	***P*** ^$^	Placebo Mean ± SE (CI)	Vitamin D Mean ± SE (CI)	***P*** ^¶^
25(OH) vitamin D (ng/mL) (Crude)	31.4 ± 8.4 (27.5, 35,4)	31.7 ± 10.3 (27.8, 35.7)	0.91	27.5 ± 8.7 (21.7,33.3)	46.9 ± 17.6 (41.1,52.8)	< 0.001	-3.9 ± 10.9 (-9.1,1.1)	15.2 ± 13.7 (10.1,20.4)	< 0.001
(Adjusted)	-	-	-	27.7 ± 12.4 (22.3, 4.9)	46.8 ± 12.4 (41.5, 5.0)	< 0.001	-3.9 ± 2.6 (-9.2, 1.3)	15.1 ± 2.6 (9.9, 20.4)	< 0.001
VDR (ng/mL) (Crude)	55.5 ± 14.2 (48.9,62.2)	58.6 ± 17.3 (52.0,65.3)	0.51	53.0 ± 12.7 (47.3,58.8)	62.7 ± 14.6 (56.9,68.5)	0.04	-2.5 ± 10.5 (-7.0,0.9)	4.1 ± 11.1 (0.7,8.6)	0.008
(Adjusted)	-	-	-	54.3 ± 9.6 (50.6,3.2)	62.6 ± 9.6 (57.6,5.4)	0.01	-2.8 ± 2.0 (-6.8,1.2)	4.4 ± 2.0 (0.4,8.4)	0.01
PTH (ng/mL) (Crude)	34.6 ± 8.8 (31.2,38.0)	34.6 ± 7.4 (31.2,38.0)	0.99	22.5 ± 7.7 (19.7,25.2)	19.3 ± 5.3 (16.5,22.1)	0.28	-12.1 ± 11.8 (-15.0,-9.4)	-15.3 ± 7.4 (-18.1,12.5)	0.11
(Adjusted)	-	-	-	22.3 ± 6.7 (19.25,5.1)	19.5 ± 6.7 (16.22,7.3)	0.17	-12.4 ± 1.4 (-15.2,-9.5)	-15.2 ± 1.4 (-18.0,-12.3)	0.17
ALT (IU/L) (Crude)	30.3 ± 24.1 (19.7,40.8)	33.9 ± 26.1 (23.3, 44.5)	0.62	36.2 ± 25.1 (26.9,45.4)	22.1 ± 18.3 (12.9,31.4)	< 0.001	5.9 ± 13.4 (0.4,10.4)	-11.8 ± 13.5 (-16.3,-6.3)	< 0.001
(Adjusted)	-	-	-	37.6 ± 12.0 (32.5,42.7)	20.7 ± 12.0 (15.6,25.8)	< 0.001	5.5 ± 2.5 (0.4,10.6)	-11.4 ± 2.5 (-16.5,-6.3)	< 0.001
AST (IU/L) (crude)	24.2 ± 9.2 (19.8,28.7)	25.3 ± 11.8 (20.8, 29.7)	0.73	25.4 ± 9.6 (21.5,29.2)	20.9 ± 8.7 (17.1,24.8)	0.001	1.2 ± 3.9 (-1.0,3.0)	-4.3 ± 6.6 (-6.2,-2.2)	0.001
(Adjusted)	-	-	-	25.7 ± 4.8 (23.7,27.7)	20.6 ± 4.8 (18.6,22.7)	0.001	0.94 ± 1.0 (-1.1, 3.0)	-4.1 ± 1.0 (-6.1,-2.1)	0.001
Fasting serum glucose (mg/dL) (Crude)	102.0 ± 21.9 (93.7, 110.3))	103.9 ± 17.3 (95.6,112.2)	0.74	108.9 ± 24.8 (100.1,117.6)	98.7 ± 15.8 (89.9,107.4)	< 0.001	6.9 ± 9.4 (3.6,10.1)	-5.2 ± 5.1 (-8.4,-2.0)	< 0.001
(Adjusted)	-	-	-	109.7 ± 7.7 (106.4,113.0)	97.9 ± 7.7 (94.6,101.1)	< 0.001	6.7 ± 1.6 (3.5,10.0)	-5.1 ± 1.6 (-8.4,-1.8)	< 0.001
Fasting serum insulin (µIU/mL) (Crude)	24.6 ± 9.5 (20.8,28.3)	25.2 ± 8.2 (21.5,29.0)	0.81	21.8 ± 10.4 (16.8,26.7)	24.8 ± 13.1 (19.8,29.8)	0.54	-2.8 ± 11.5 (-8.0,1.9)	-0.44 ± 14.8 (-5.1,4.7)	0.41
(Adjusted)	-	-	-	21.6 ± 12.0 (16.7,26.7)	24.8 ± 12.0 (19.8,29.8)	0.38	-3.2 ± 2.5 (-8.2,1.8)	-0.1 ± 2.5 (-5.1,4.9)	0.38
HOMA-IR (Crude)	6.1 ± 2.6 (5.2,7.1)	6.4 ± 1.9 (5.4,7.4)	0.69	5.8 ± 2.9 (4.4,7.2)	6.1 ± 3.7 (4.7,7.5)	0.99	-0.29 ± 3.1 (-1.7,1.0)	-0.29 ± 3.8 (-1.6,1.1)	0.87
(Adjusted)	-	-	-	5.8 ± 3.4 (4.5,7.3)	6.1 ± 3.4 (4.7,7.4)	0.87	-0.37 ± 0.69	-0.21 ± 0.69	0.87
QUICKI (Crude)	0.30 ± 0.01 (0.29,0.30)	0.29 ± 0.01 (0.29,0.31)	0.53	0.30 ± 0.02 (0.29,0.31)	0.30 ± 0.02 (0.29,0.31)	0.88	0.006 ± 0.0 (-0.003,0.02)	0.007 ± 0.02 (-0.003,0.02)	0.99
(Adjusted)	-	-	-	0.30 ± 0.02 (0.29,0.31)	0.30 ± 0.02 (0.29,0.31)	0.91	0.007 ± 0.00 (-0.003,0.02)	0.006 ± 0.005 (-0.004,0.02)	0.91
HDL (mg/dL) (Crude)	47.6 ± 10.1 (42.3,52.9)	47.3 ± 14.5 (42.0,52.6)	0.93	45.6 ± 7.8 (41.0,50.2)	51.3 ± 13.4 (46.6,55.9)	< 0.001	-2.0 ± 5.2 (-3.9,-0.006)	3.9 ± 5.4 (2.0,5.9)	< 0.001
(Adjusted)	-	-	-	45.5 ± 4.8 (43.5,47.5)	51.3 ± 4.8 (49.3,53.3)	< 0.001	-1.9 ± 1.0 (-3.9,0.08)	3.9 ± 1.0 (1.9, 5.9)	< 0.001
LDL (mg/dL) (Crude)	100.2 ± 22.8 (89.6,110.8)	103.8 ± 27.4 (93.2,114.4)	0.63	105.9 ± 24.4 (94.6,117.2)	94.8 ± 29.1 (83.5,106.1)	0.008	5.7 ± 19.5 (-2.0,12.8)	-9.0 ± 16.6 (-16.1,-1.3)	0.01
(Adjusted)	-	-	-	106.0 ± 16.3 (99.0,113.0)	94.7 ± 16.3 (87.7,101.6)	0.03	4.0 ± 3.4 (-2.9,11.0)	-7.3 ± 3.4 (-14.3,-0.36)	0.03
TC(mg/dL) (Crude)	188.8 ± 34.3 (172.4,205.1)	194.1 ± 43.1 (177.7,210.4)	0.65	198.8 ± 36.0 (181.4,216.2)	183.6 ± 46.1 (166.2,200.1)	0.02	10.0 ± 23.4 (-2.8,21.6)	-10.5 ± 35.6 (-22.1,2.3)	0.03
(Adjusted)	-	-	-	197.7 ± 27.3 (187.1,210.3)	183.7 ± 27.3 (172.1,195.3)	0.07	7.2 ± 5.7 (-4.3,18.8)	-7.7 ± 5.4 (-19.3,3.8)	0.07
TG ((mg/dL) Crude)	153.1 ± 85.8 (121.5,184.6)	143.6 ± 62.6 (112.0,175.2)	0.67	161.0 ± 93.2 (127.3,194.8)	138.2 ± 65.0 (104.4,172.0)	0.25	7.9 ± 38.4 (-8.3,24.8)	-5.4 ± 39.9 (-22.2,10.9)	0.24
(Adjusted)	-	-	-	155.9 ± 39.8 (139.1,172.7)	143.3 ± 39.8 (126.5,160.1)	0.29	7.6 ± 8.3 (-9.2,24.4)	-5.0 ± 8.3 (-21.8,11.8)	0.29

^*^Obtained by Independent sample t-test

^$^ Obtained by Repeated measure ANOVA

^¶^ Obtained by ANCOVA

Despite a non-significant difference between the two group in terms of ALT, AST, FBS and LDL-C at study baseline, 12-week vitamin D supplementation led to significant reductions in serum levels of ALT (changes from baseline in vitamin D group: -11.8 mg/dL vs. 5.9 mg/dL in placebo group, *P* < 0.001), AST (-4.3 mg/dL vs. 1.2 mg/dL, *P* = 0.001), FBS (-5.2 mg/dL vs. 6.9 mg/dL, *P* < 0.001), LDL-C (-9.0 mg/dL vs. 5.7 mg/dL, *P* = 0.01), and TC (-10.5 mg/dL vs. 10.0 mg/dL, *P* = 0.03) compared with placebo. In an effort to remove the confounding effect of baseline levels and medications use, an additional analysis was conducted. We found that after controlling for these variables, all above-mentioned effects remained significant except for TC (-7.7 mg/dL vs. 7.2 mg/dL, *P* > 0.05).

There were no significant differences between the two groups concerning other metabolic variables at study end (*P* > 0.05).

Table 4 shows the fibrogenic factors of patients at study baseline and after 12 weeks of intervention. Although there was no significant difference between the vitamin D and placebo groups in terms of laminin and collagen type IV (*P* > 0.05), serum hyaluronic acid was significantly different between the two groups at study baseline (*P* = 0.04). Supplementation with vitamin D for 12 weeks resulted in a significant decrease in serum laminin concentrations (changes from baseline in vitamin D group: -10.6 ng/mL vs. 4.9 ng/mL in placebo group, *P* = 0.01) and hyaluronic acid (-28.7 ng/mL vs. -3.5 ng/mL, *P* = 0.04) compared to placebo. Serum levels of collagen type IV was not significantly different between the two groups at the endpoint (-26.5 ng/mL vs. -8.5 ng/mL, *P* = 0.09). These findings did not change after controlling for baseline levels.

**Table 4:** Fibrogenic factors of study participants at base line and after 12 weeks trial

	Base line			End of trial			Changes from baseline		
	Placebo Mean ± SD (CI)	Vitamin D Mean ± SD (CI)	***P*** ^*^	Placebo Mean ± SD (CI)	Vitamin D Mean ± SD (CI)	***P*** ^$^	Placebo Mean ± SE (CI)	Vitamin D Mean ± SE (CI)	***P*** ^¶^
Laminin (ng/mL) (Crude)	73.6 ± 20.3 (66.0,81.3)	71.5 ± 15.6 (63.8,79.1)	0.69	78.6 ± 29.3 (68.0,89.1)	60.9 ± 20.1 (50.3,71.5)	0.01	5.1 ± 4.1 (-3.3,13.4)	-10.7 ± 4.1 (-19.1,-2.3)	0.01
(Adjusted)	-	-	-	77.5 ± 20.1 (69.0,86.0)	61.9 ± 20.1 (53.4,70.5)	0.01	4.9 ± 4.2 (-3.6,13.5)	-10.6 ± 4.2 (-19.1,-2.1)	0.01
Hyaluronic acid (ng/mL) (Crude)	106.7 ± 22.7 (96.2,117.2)	121.8 ± 26.9 (111.4,132.3)	0.04	103.2 ± 35.0 (91.0,115.4)	93.1 ± 21.1 (80.9,105.2)	0.005	-3.5 ± 34.5 (-18.7,4.1)	-28.7 ± 22.1 (-36.3,13.5)	0.04
(Adjusted)	-	-	-	107.2 ± 27.3 (95.6,118.8)	89.1 ± 27.3 (77.4,100.7)	0.04	-7.1 ± 5.7 (-18.7,4.6)	-25.2 ± 5.7 (-36.8,13.6)	0.03
Collagen type IV (ng/mL) (Crude)	264.6 ± 34.8 (246.1,283.1)	264.4 ± 51.7 (245.9,282.9)	0.99	256.1 ± 33.4 (236.5,275.6)	237.9 ± 56.7 (218.3,257.4)	0.11	-8.5 ± 38.6 (-23.3,6.3)	-26.5 ± 35.7 (-41.3,11.7)	0.09
(Adjusted)	-	-	-	254.8 ± 35.2 (240.0,269.7)	239.1 ± 35.2 (224.3,254.0)	0.14	-9.7 ± 7.3 (-24.5,5.1)	-25.4 ± 7.3 (-40.2,10.5)	0.14

^*^Obtained by Independent sample t-test

^$^ Obtained by Repeated measure ANOVA.

^¶^ Obtained by ANCOVA.

Mean fold changes of MicroRNAs of participants at study baseline and after 12 weeks of intervention are provided in Table 5. At study baseline, there was no significant mean difference between the vitamin D and placebo groups in terms of all MicroRNAs (*P* > 0.05). Supplementation with vitamin D for 12 weeks led to a significant lower MiR-21 gene expression compared to the placebo group at week 12 (changes from baseline in vitamin D group: 0.16 RFC vs. 1.49 RFC in placebo group, *P* = 0.01). MiR-122s gene expression significantly decreased following the vitamin D supplementation (-0.77 RFC vs. 0.93 RFC, *P* < 0.001) compared with placebo. Gene expression of MiR-34a was not significantly different between the two groups at study endpoint (0.05 RFC vs. 0.05 RFC, *P* = 0.22). These findings persisted after adjustment for potential confounders.

**Table 5:** The mean of fold changes of MicroRNAs of study participants at base line and after 12 weeks trial

	Baseline			End of trial			Changes from baseline		
	Placebo Mean ± SD (CI)	Vitamin D Mean ± SD (CI)	***P*** ^*^	Placebo Mean ± SD (CI)	Vitamin D Mean ± SD (CI)	***P*** ^$^	Placebo Mean ± SE (CI)	Vitamin D Mean ± SE (CI)	***P*** ^¶^
MiR-21 (Crude)	1.50 ± 1.64 (0.95,2.04)	1.50 ± 0.81 (0.96,2.05)	0.99	3.00 ± 1.80 (2.3,3.7)	1.67 ± 1.60 (0.95,2.4)	0.03	1.49 ± 2.10 (0.78,2.2)	0.16 ± 1.82 (-0.55,0.88)	0.01
(Adjusted)	-	-	-	3.01 ± 1.7 (2.3,3.7)	1.65 ± 1.7 (0.92,2.4)	0.01	1.5 ± 0.36 (0.78,2.2)	0.15 ± 0.36 (-0.58,0.87)	0.01
MiR-122 (Crude)	1.19 ± 1.34 (0.68,1.7)	1.53 ± 1.1 (1.0,2.0)	0.35	2.11 ± 1.75 (1.5,2.7)	0.75 ± 0.67 (0.2,1.3)	< 0.001	0.93 ± 1.26 (0.42,1.3)	-0.77 ± 0.98 (-1.2,-0.26)	< 0.001
(Adjusted)	-	-	-	2.21 ± 1.05 (1.7,2.7)	0.65 ± 1.05 (0.2,1.1)	< 0.001	0.86 ± 0.23 (0.40,1.31)	-0.70 ± 0.23 (-1.1,-0.25)	< 0.001
MiR-34a (Crude)	1.48 ± 1.03 (1.1,1.9)	1.02 ± 0.86 (0.63,1.4)	0.11	1.54 ± 1.00 (1.1,1.9)	1.07 ± 0.94 (0.7,1.5)	0.98	0.05 ± 1.09 (-0.18,0.64)	0.05 ± 1.30 (-0.54,0.28)	0.22
(Adjusted)	-	-	-	1.49 ± 0.96 (1.1,1.9)	1.12 ± 0.96 (0.7,1.5)	0.22	0.24 ± 0.21 (-0.18,0.66)	-0.14 ± 0.21 (-0.56,0.28)	0.22

^*^Obtained by Independent sample t-test

^$^ Obtained by Repeated measure ANOVA

^¶^ Obtained by ANCOVA

We also performed per-protocol analyses for all serum metabolic profile, fibrogenic factors and MicroRNAs. However, results were not changed after these analyses. (data are shown in supplementary material 1).

There were significant inverse correlations between changes in serum concentrations of vitamin D with laminin and hyaluronic acid (*r*= -0.294 and *r*= -0.290; respectively, *p* < 0.05). This correlation between changes in serum levels of vitamin D and collagen type IV was not significant (*r*= -0.032, *p* > 0.05). In case of serum MiRs, we found slight inverse significant correlations between changes in serum vitamin D levels with MiR-21 and MiR-122 gene expression changes in our study population (*r*= -0.288 and *r*= -0.284; respectively, *p* = 0.05). However, there was no significant correlation between serum vitamin D change and changes in gene expression of MiR-34a (*r*= -0.085, *p* > 0.05).

## Discussion

In this randomised, placebo controlled clinical trial on patients with metabolic dysfunction-associated steatotic liver disease (MASLD), 4000 IU/daily vitamin D supplementation for 12 weeks resulted in a significant increased levels of serum 25(OH)D_3_, VDR and HDL-C, and to decreased serum concentrations of ALT, AST, FBS and LDL-C as well as serum fibrogenic factors including laminin and hyaluronic acid. We found a significant difference between the two groups in terms of serum MiR-21 and MiR-122 at the end of trial. To the best of our knowledge, the present study is the first to investigate the effectiveness of 4000 IU/daily vitamin D supplementation on liver fibrogenic factors and liver fibrosis-related microRNAs in MASLD patients.

In addition to the roles of vitamin D on calcium haemostasis and bone health, it functions as an anti-inflammatory agent [30] and can modulate gene expression of specific genes involved in energy and lipid metabolism as well as insulin haemostasis [15]. Vitamin D mediates the physiologic roles via its specific intra-cellular receptor receptor (VDR) [15, 31, 32]. Lower levels of vitamin D are associated with hepatic steatosis, necroinflammation and liver fibrosis [15, 21, 31, 32]. Treatment of hepatic stellate cells (HSCs) with 6–10 M vitamin D_2_ for 24 h, significantly suppresses the gene expression of profibrogenic factors (COL1α, α-SMA, TGF-β) and inflammatory cytokines [33]. Similarly, calciteriol inhibited TGF-β1-induced collagen type I and III and hyaluronan synthesis in human dermal fibroblast in both in vitro and in vivo situations [34]. Intraperitoneal injections of 1, 5, or 10 µg/kg of 1,25-vitmain D3 twice weekly for 12 weeks to Wistar rats with NASH decreased the collagen type I and liver fibrosis [35]. Findings of the present clinical trial showed that vitamin D supplementation for 12 weeks significantly reduced circulating levels of laminin and hyaluronic acid and non-significantly for collagen type IV level compared to placebo. A significant inverse correlation was also seen between changes in fibrogenic factors and changes in serum vitamin D levels, except for collagen type IV, where the correlation was seen only in the intervention group. It may be caused by secretion of collagens from other tissues including bone and cartilage [36]; and consequently disrupt the serum levels of collagens in MASLD patients. However, as we excluded patients with confirmed co-morbidities including any chronic disease, it is less likely possible to significantly affect the collagen type IV levels by secretion of other tissues.

The most important signaling pathway in the HSCs which contributes to liver fibrosis is TGF-β/SMAD signaling pathway [37]. Activation of this cascade leads to production of various fibrogenic factors including collagens, laminin and other proteins of cellular matrix in HSCs [38]. As a result, some fibrotic agents including hyaluronic acid deposits in the extra-cellular matrix [38, 39]. Vitamin D and its receptor specifically antagonize this signaling pathway in the liver. Animal studies have found that VDR-knockout mice develop liver fibrosis [4, 15, 39]. Moreover, vitamin D/VDR complex increases the gene expression of anti-fibrotic proteins such as Bone Morphogenetic Protein 7 (BMP 7) and matrix metalloproteinase 8 (MMP 8) [40]. In addition, this complex can suppress the Ras/ERK-P signaling pathways and consequently inhibits the HSCs proliferation and decrease liver fibrosis [15]. Finally, some studies have shown that vitamin D decreases the production and activation of other fibrogenic factors including plasminogen activator inhibitor-1 (SERPINE1) [39].

We found that vitamin D consumption for 12 weeks suppressed the gene expression of pro-fibrogenic MiR-21 levels and the gene expression of anti-fibrogenic MiR-122 but not pro-fibrogenic MiR-34a compared to placebo. Although the overall gene expression of MiR-21 increased in both groups, the elevation in placebo group was significantly higher than in the vitamin D group. In a previous clinical trial, daily supplementation with 2000–4000 IU vitamin D for 12 months had no significant effects on serum levels of MiR-221 in healthy men [40]. Among athletes, a single dose of 10,000 IU vitamin D resulted in a significant decreased serum levels of pro-inflammatory MiRs; however, serum levels of MiR-21 did not change compared to baseline [41]. Of note, the levels of liver fibrosis-related MiRs are affected by the secretions of these factors from tissues [42, 43]. It has been shown that TGF-β signaling is at the heart of fibrotic signaling pathways in the liver and is modulated by networks of pro- and anti-fibrotic MiRs. Therefore, anti-fibrotic effects of vitamin D in patients with MASLD can be mediated by TGF-β signaling [17, 44]. We did not examine the effects of vitamin D on the gene expression of TGF-β, but consumption of vitamin D_2_ with doses of 60,000, 80,000, 100,000 IU/weekly for vitamin D insufficient, deficient, severely insufficient in patients with chronic hepatitis C for 6 weeks has no significant effect on serum TGF-β1, TIMP-1, MMP-9 and P3NP compared to placebo group [45].

Some clinical trials examined vitamin D supplementation on non-serum biomarkers of liver fibrosis. 1000 IU/daily vitamin D intake for 360 days decreased indices of liver steatosis and fibrosis assessed by transient elastography (TE, FibroScan®) in comparison to placebo [46]. By contrast, another report did not show a significant effect of vitamin D_3_ consumption (2100 IU/day for 48 weeks) on hepatic steatosis, possibly due to limited number of available biopsy specimens [47]. The type, dose and duration of vitamin D supplement, characteristics of the study population, and sample size may contribute to apparently conflicting results.

Our findings indicated that vitamin D supplementation can increase the serum levels of 25(OH) vitamin D, VDR and HDL-C, and led to significant decrease in liver enzymes levels including ALT and AST as well as FBS and LDL-C. However, this supplementation did not influence the serum concentration of PTH, insulin, HOMA-IR, QUICKI, TG and TC. Our findings were in accord with others that indicated that vitamin D supplementation increased the serum levels of VDR but did not affect the PTH [48, 49]. By contrast, in one study, after injection of a 600,000 IU vitamin D IM, serum PTH levels significantly increased in placebo group [50]. Also, supplementation with 20,000 IU/week of vitamin D for 6 weeks significantly decreased the serum PTH compared to placebo [51]. It is interesting that serum PTH levels went down in vitamin D group. Maybe people were already pretty high in 25(OH) vitamin D levels for vitamin D to have an effect on PTH. Usually, the effect is when people are vitamin D deficient. In non-obese subjects, vitamin D supplementation decreased the PTH levels in a linear manner, but in obese individuals, PTH levels remained 10 pg/ml higher than non-obese subjects after 3-month supplementation with 2000 to 4000 IU/ day vitamin D [52]. Possibly, in obese MASLD patients’ vitamin D supplementation even in higher levels without significant changes in PTH levels may affect the liver [52].

Regarding liver enzymes, in line with present study, some clinical trials have reported a significant reduction in serum ALT and AST levels [47, 53, 54]. By contrast, others have found significant decrease only in ALT [55]. Some studies failed to find significant effect on serum transaminases by vitamin D supplementation [51, 55]. Considering metabolic profile including glycemic factors and lipid profile have shown that similar to our findings, some previous studies have reported a significant decrease in LDL-C and a significant increase in HDL-C levels after vitamin D consumption [23]. Other clinical trials have reached significant results only in serum TG or TC concentrations [54]. In line with our results, some studies failed to find significant effects on serum insulin and HOMA-IR with a significant reduction in fasting blood sugar by vitamin D treatment [20–23]. By contrast, some others have shown a significant reduction in serum insulin and HOMA-IR without any changes in blood glucose [50, 54]. Zanko et al. showed a significant reduction in fasting serum levels of insulin, HOMA-IR and gamma glutamyl transferase (γGT) with daily consumption of 1000 IU vitamin D for 360 days [46]. Recent meta-analysis concluded that vitamin D supplementation has no significant effect on reduction of ALT, AST and γGT but suggested a significant reduction in alkaline phosphatise levels [56]. However, dose-response analyses were not done in the meta-analysis. Conflicting results may be related to the dose and duration of vitamin D supplementation, and to metabolic status of subjects [48].

Being the first report on the effects of vitamin D supplementation on liver fibrosis-related fibrogenic factors and MiRs, using the Taqman assay method, as well as using a block randomized, placebo-controlled design are strengths of the present clinical trial. However, some limitations should be considered in interpretation of our findings. We did not use the gold standard method for evaluating NASH, which is the liver histopathology. Despite the highest accuracy, this invasive method is not routine. We used other non-invasive and routine methods including B-ultrasound and liver fibroscan to evaluate NASH. The sample size of study is relatively small, and due to funding limitations, we were unable to measure the gene expression of TGF/β. Gene expression of other fibrogenic factors including MMPs and other MiRs in particular anti-fibrogenic MiRs and endpoint liver fibroscan were not measured. The vitamin D supplementation in present study was set on upper limit, 4000 IU/daily for 12 weeks, which is more than the standard recommendations. This protocol is different from the other supplementation settings that considered for MASLD patients to examine the lipid profile, glycemic indices and hepatic enzymes. Some studies have implemented the 50,000 IU/weekly doses of vitamin D supplement in different durations [20, 22, 23]. However, based on our literature review, obese patients have increased daily needs of vitamin D [57]. Moreover, it has been suggested that high daily doses of vitamin D supplement is more physiologic than weekly supplementation [58]. Although we applied the upper limits of vitamin D supplementation and high compliance rate, we achieved serum 25 (OH) vitamin D levels of 46.9 ng/mL, which are not excessive.

In conclusion, we found a significant reduction in some liver fibrogenic factors and in liver transaminases and corresponding changes in some fibrosis-related MiRs and some metabolic factors by a 12-week vitamin D supplementation in MASLD patients. However, further clinical trials with larger sample sizes and direct measures of liver fibrosis are needed to confirm these findings.

### Electronic supplementary material

Below is the link to the electronic supplementary material.

Supplementary Material 1

Supplementary Material 2

Supplementary Material 3

## Data Availability

Data sharing was not applicable to this study.
