# Supplementary material for: Effects of vitamin D supplementation on liver fibrogenic factors, vitamin D receptor and liver fibrogenic microRNAs in metabolic dysfunction-associated steatotic liver disease (MASLD) patients: an exploratory randomized clinical trial
Source: Nutr J. 2024 Feb 27;23:24. doi: 10.1186/s12937-024-00911-x (PMC10898146; doi:10.1186/s12937-024-00911-x)
Supplement: Supplementary file 1 — Supplementary Material 1 [file 12937_2024_911_MOESM1_ESM.docx]

**Table 1.** Metabolic status of study participants at baseline and after 12 weeks trial: per- protocol analyses

|  | Study Baseline | | | End of trial | | | Changes from baseline | | |
| --- | --- | --- | --- | --- | --- | --- | --- | --- | --- |
|  | Placebo  Mean±SD  (CI) | Vitamin D  Mean±SD  (CI) | P^*^ | Placebo  Mean±SD  (CI) | Vitamin D  Mean±SD  (CI) | P^$^ | Placebo  Mean±SE  (CI) | Vitamin D  Mean±SE  (CI) | P^¶^ |
| 25 (OH) Vitamin D (ng/mL) (Crude) | 31.4± 8.4  (27.5, 35,4) | 31.7±10.3  (27.8, 35.7) | 0.91 | 29.54±2.3  (29.1,35.1) | 39.83±2.2  (32.8,41.7) | 0.003 | -5.5±2.7  (-11.0,-0.08) | 15.9±2.6  (10.6,21.1) | <0.001 |
| (Adjusted) | - | - | - | 26.8±9.0  (25.3, 34.6) | 47.8±17.5  (35.0, 43.9) | 0.005 | -5.4±2.8  (-11.0,-0.18) | 15.7±2.6  (10.6,21.1) | <0.001 |
| VDR (ng/mL) (Crude) | 55.5±14.2  (48.9,62.2) | 58.6±17.3  (52.0,65.3) | 0.51 | 50.9±11.4  (44.9, 56.9) | 63.2±14.8  (57.5, 68.9) | 0.04 | -2.9±2.5  (-8.0,2.2) | 5.0±2.0  (-0.6,9.1) | 0.04 |
| (Adjusted) | - | - | - | 50.9±11.4  (44.8,50.0) | 63.2±14.8  (57.4,60.9) | 0.04 | -3.6±2.1  (-7.9,0.7) | 5.0±2.0  (0.8,9.1) | 0.007 |
| PTH (ng/mL) (Crude) | 34.6±8.8  (31.2,38.0) | 34.6±7.4  (31.2,38.0) | 0.99 | 20.8±5.3  (18.6,23.0) | 18.6±4.3  (16.5,20.7) | 0.42 | -14.0±2.1  (-18.2,-9.8) | -16.0±2.0  (-20.1,-12.0) | 0.49 |
| (Adjusted) | - | - | - | 22.3±6.7  (19.25,5.1) | 19.5±6.7  (16.22,7.3) | 0.17 | -13.9±1.1  (-16.1,-11.6) | -16.2±1.1  (-18.3,-14.0) | 0.15 |
| ALT (IU/L) (Crude) | 30.3±24.1  (19.7,40.8) | 33.9±26.1  (23.3, 44.5) | 0.62 | 39.2±25.1  (29.3,49.2) | 22.4±18.7  (13.0, 31.9) | <0.001 | 6.8±3.1  (0.53,13.1) | -12.3±2.9  (-18.3,-6.3) | <0.001 |
| (Adjusted) | - | - | - | 39.2±25.1  (29.5,49.9) | 22.4±18.7  (12.3,31.7) | <0.001 | 6.8±2.7  (1.2,12.4) | -12.3±2.6  (-17.6,-7.0) | <0.001 |
| AST (IU/L) (crude) | 24.2±9.2  (19.8,28.7) | 25.3±11.8  (20.8, 29.7) | 0.73 | 26.6±9.9  (22.4,30.8) | 21.0±8.9  (17.0,251) | 0.002 | 1.3±1.2  (-1.2,3.9) | -4.5±1.2  (-7.0,-2.1) | 0.002 |
| (Adjusted) | - | - | - | 26.6±9.8  (22.5,31.2) | 21.0±8.9  (16.7,24.9) | 0.004 | 1.3±1.1  (-1.1,3.5) | -4.4±1.1  (-6.6,-2.2) | 0.001 |
| Fasting serum glucose (mg/dL) (Crude) | 102.0±21.9  (93.7, 110.3)) | 103.9±17.3  (95.6,112.2) | 0.74 | 110.7±26.1  (101.0,120.3) | 99.3±15.1  (90.1,108.5) | <0.001 | 7.9±1.7  (4.4,11.4) | -5.4±1.6  (-8.7,-2.1) | <0.001 |
| (Adjusted) | - | - | - | 110.7±26.1  (102.2,121.6) | 99.3±15.8  (89.0,107.4) | <0.001 | 7.8±1.8  (4.2,11.4) | -5.4±1.7  (-8.8,-1.9) | <0.001 |
| Fasting serum insulin (µIU/mL) (Crude) | 24.6±9.5  (20.8,28.3) | 25.2±8.2  (21.5,29.0) | 0.81 | 21.8±11.2  (16.2,27.4) | 24.6±13.3  (19.2,29.9) | 0.52 | -3.2±3.1  (-9.5,3.0) | -0.45±3.0  (-6.4,5.5) | 0.52 |
| (Adjusted) | - | - | - | 21.8±11.2  (15.9,27.4) | 24.6±13.3  (19.2,30.1) | 0.50 | -3.4±2.8  (-9.0,2.3) | -0.36±2.7  (-5.8,5.0) | 0.52 |
| HOMA-IR (Crude) | 6.1±2.6  (5.2,7.1) | 6.4±1.9  (5.4,7.4) | 0.69 | 5.9±3.2  (4.3,7.5) | 6.1±3.8  (4.6,7.6) | 0.99 | -0.30±0.81  (-1.9,1.3) | -0.30±0.77  (-1.88,1.3) | 0.99 |
| (Adjusted) | - | - | - | 5.9±3.2  (4.3,7.5) | 6.1±3.8  (4.5,7.6) | 0.98 | -0.35±0.78  (-1.9,1.2) | -0.26±0.74  (-1.7,1.2) | 0.93 |
| QUICKI (Crude) | 0.30±0.01  (0.29,0.30) | 0.29±0.01  (0.29,0.31) | 0.53 | 0.30±0.03  (0.29,0.32) | 0.30±0.02  (0.29,0.31) | 0.94 | 0.007±0.006  (-0.005,0.02) | 0.008±0.005  (-0.003,0.02) | 0.93 |
| (Adjusted) | - | - | - | 0.30±0.03  (0.29,0.32) | 0.30±0.02  (0.29,0.31) | 0.94 | 0.007±0.006  (-0.004,0.02) | 0.008±0.005  (-0.004,0.02) | 0.89 |
| HDL (mg/dL) (Crude) | 47.6±10.1  (42.3,52.9) | 47.3±14.5  (42.0,52.6) | 0.93 | 45.4±8.0  (40.2,50.3) | 50.2±12.8  (45.6,55.2) | 0.001 | -2.3±1.2  (-4.8,0.2) | 4.1±1.2  (1.8,6.5) | <0.001 |
| (Adjusted) | - | - | - | 45.4±8.0  (40.2,50.3) | 50.2±12.8  (45.6,55.2) | 0.001 | -2.0±1.1  (-4.3,0.2) | 3.9±1.0  (1.7,6.0) | <0.001 |
| LDL (mg/dL) (Crude) | 100.2±22.8  (89.6,110.8) | 103.8±27.4  (93.2,114.4) | 0.63 | 105.7±22.5  (93.7,117.7) | 94.2±29.7  (82.7,105.6) | 0.009 | 6.6±4.2  (-1.9,15.1) | -9.4±4.0  (-17.6,-1.3) | 0.009 |
| (Adjusted) | - | - | - | 105.7±22.5  (91.7,114.3) | 94.1±29.7  (85.8,107.3) | 0.02 | 4.2±3.9  (-3.7,12.1) | -7.2±3.7  (-14.8,-0.3) | 0.04 |
| TC(mg/dL) (Crude) | 188.8±34.3  (172.4,205.1) | 194.1±43.1  (177.7,210.4) | 0.65 | 198.2±32.0  (180.0,216.3) | 181.7±46.4  (164.4,199) | 0.02 | 11.5±7.0  (-2.6,25.7) | -11±6.7  (-24.5,2.5) | 0.02 |
| (Adjusted) | - | - | - | 198.2±32.0  (177.0,211.4) | 181.7±46.4  (169.0,201.6) | 0.06 | 7.8±6.5  (-5.3,20.9) | -7.6±6.2  (-20.1,4.9) | 0.1 |
| TG ((mg/dL) Crude) | 153.1±85.8  (121.5,184.6) | 143.6±62.6  (112.0,175.2) | 0.67 | 153.3±83.8  (119.5,187.1) | 140.4±65.7  (108.2,172.7) | 0.25 | 9.1±9.1  (-9.4,27.7) | -5.6±8.7  (-23.3,12.0) | 0.25 |
| (Adjusted) | - | - | - | 153.3±83.8  (117.8,187.4) | 140.4±65.7  (107.9,174.2) | 0.33 | 8.2±9.4  (-10.9,27.3) | -4.8±9.0  (-22.9,13.4) | 0.33 |

^*^Obtained by Independent sample t-test.

^$^ Obtained by Repeated measure ANOVA.

^¶^ Obtained by ANCOVA.

**Table 2.** Fibrogenic factors of study participants at base line and after 12 weeks trial: per- protocol analyses

|  | Base line | | | End of trial | | | Changes from baseline | | |
| --- | --- | --- | --- | --- | --- | --- | --- | --- | --- |
|  | Placebo  Mean±SD  (CI) | Vitamin D  Mean±SD  (CI) | P^*^ | Placebo  Mean±SD  (CI) | Vitamin D  Mean±SD  (CI) | P^$^ | Placebo  Mean±SE  (CI) | Vitamin D  Mean±SE  (CI) | P^¶^ |
| Laminin (ng/mL) (Crude) | 73.6±20.3  (66.0,81.3) | 71.5±15.6  (63.8,79.1) | 0.69 | 77.7±29.9  (66.2,89.2) | 61.0±20.6  (50.0,71.9) | 0.01 | 5.3±4.6  (-4.0,14.7) | -11.0±4.4  (-20.0,-1.9) | 0.01 |
| (Adjusted) | - | - | - | 77.7±29.9  (65.6,89.1) | 61.0±20.6  (50.1,72.4) | 0.01 | 5.3±4.7  (-4.3,14.9) | -11.0±4.5  (-20.2,-1.9) | 0.01 |
| Hyaluronic acid (ng/mL) (Crude) | 106.7±22.7  (96.2,117.2) | 121.8±26.9  (111.4,132.3) | 0.04 | 101.2±36.2  (88.9,114.5) | 92.6±21.5  (79.9,105.3) | 0.007 | -3.7±6.7  (-17.3,9.9) | -30.0±6.4  (-43.0,17.1) | 0.007 |
| (Adjusted) | - | - | - | 101.2±36.2  (88.1,115.2) | 92.6±21.5  (79.2,105.0) | 0.006 | -8.1±6.4  (-21.1,4.9) | -26.1±6.1  (-3.4,13.7) | 0.05 |
| Collagen type IV (ng/mL) (Crude) | 264.6±34.8  (246.1,283.1) | 264.4±51.7  (245.9,282.9) | 0.99 | 255.5±34.2  (233.7,277.2) | 237.9±58.0  (217.1,258.7) | 0.15 | -9.0±8.7  (-26.7,8.6) | -26.5±8.3  (-43.3,-9.7) | 0.15 |
| (Adjusted) | - | - | - | 255.5±34.2  (231.4,274.1) | 237.9±58.0  (220.0,260.7) | 0.20 | -10.4±8.3  (-27.2,6.4) | -25.3±7.9  (-41.2,-9.3) | 0.21 |

^*^Obtained by Independent sample t-test.

^$^ Obtained by Repeated measure ANOVA.

^¶^ Obtained by ANCOVA.

**Table 3.** The mean of fold changes of MicroRNAs of study participants at base line and after 12 weeks trial: per- protocol analyses

|  | Baseline | | | End of trial | | | Changes from baseline | | |
| --- | --- | --- | --- | --- | --- | --- | --- | --- | --- |
|  | Placebo  Mean±SD  (CI) | Vitamin D  Mean±SD  (CI) | P^*^ | Placebo  Mean±SD  (CI) | Vitamin D  Mean±SD  (CI) | P^$^ | Placebo  Mean±SE  (CI) | Vitamin D  Mean±SE  (CI) | P^¶^ |
| MiR-21  (Crude) | 1.50± 1.64  (0.95,2.04) | 1.50±0.81  (0.96,2.05) | 0.99 | 3.32±1.68 (2.58,4.1) | 1.72±1.61  (1.01,2.4) | 0.01 | 1.72±0.45  (0.81,2.63) | 0.17±0.43  (-0.7,1.03) | 0.01 |
| (Adjusted) | - | - | - | 3.32±1.68 (2.6,4.1) | 1.72±1.61  (0.96,2.4) | 0.01 | 1.79±0.38  (1.02,2.56) | 0.11±0.36  (-0.62,0.84) | 0.003 |
| MiR-122 (Crude) | 1.19±1.34  (0.68,1.7) | 1.53±1.1  (1.0,2.0) | 0.35 | 2.40±1.70  (1.82,2.97) | 0.77±0.69  (0.22,1.31) | <0.001 | 1.07±0.26  (0.55,1.58) | -0.81±0.24  (-1.30,-0.31) | <0.001 |
| (Adjusted) | - | - | - | 2.40±1.70  (1.85,3.03) | 0.77±0.69  (0.17,1.29) | <0.001 | 1.01±0.24  (0.53,1.50) | -0.76±0.23  (-1.22,-0.29) | <0.001 |
| MiR-34a (Crude) | 1.48±1.03  (1.1,1.9) | 1.02±0.86  (0.63,1.4) | 0.11 | 1.72±0.94  (1.29,2.14) | 1.09±0.96  (0.68,1.50) | 0.97 | 0.06±0.28  (-0.50,0.63) | 0.05±0.27  (-0.49,0.59) | 0.97 |
| (Adjusted) | - | - | - | 1.72±0.94  (1.31,2.18) | 1.09±0.96  (0.65,1.49) | 0.93 | 0.38±0.23  (-0.08,0.84) | -0.24±0.22  (-0.67,0.20) | 0.07 |

^*^Obtained by Independent sample t-test.

^$^ Obtained by Repeated measure ANOVA.

^¶^ Obtained by ANCOVA.
