# Supplementary figures and images for: Effects of vitamin D supplementation on liver fibrogenic factors, vitamin D receptor and liver fibrogenic microRNAs in metabolic dysfunction-associated steatotic liver disease (MASLD) patients: an exploratory randomized clinical trial
Source: Nutr J. 2024 Feb 27;23:24. doi: 10.1186/s12937-024-00911-x (PMC10898146; doi:10.1186/s12937-024-00911-x)

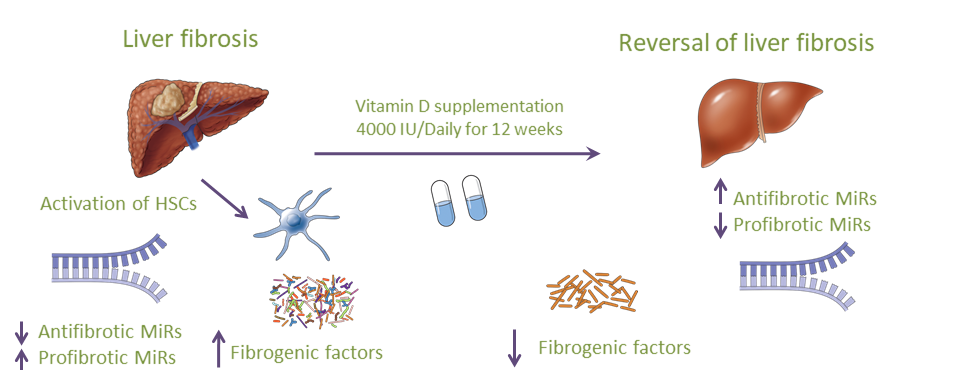

Supplement: Supplementary file 3 — Supplementary Material 3 [file 12937_2024_911_MOESM3_ESM.tif]
